# Predictive Impact of Peritoneal Computed Tomography Attenuation Values for the Severity of Upper Gastrointestinal Perforation

**DOI:** 10.31662/jmaj.2025-0189

**Published:** 2025-09-12

**Authors:** Hironori Tsujimoto, Risa Kariya, Seiichiro Fujishima, Takafumi Suzuki, Naoyuki Uehata, Yoshihisa Yaguchi, Keita Kouzu, Hiroshi Shinmoto, Hideki Ueno

**Affiliations:** 1 Department of Surgery, National Defense Medical College, Namiki, Tokorozawa, Japan; 2 Department of Radiology, National Defense Medical College, Namiki, Tokorozawa, Japan

**Keywords:** severity of the illness, multidetector CT, severity scoring system, upper gastrointestinal perforation, sepsis

## Abstract

**Introduction:**

This study aimed to evaluate the peritoneal computed tomography (CT) attenuation values and investigate their predictive impact for the severity of peritonitis in patients with upper gastrointestinal tract (UGI) perforations.

**Methods:**

Overall, 112 consecutive patients with UGI perforations who underwent plain CT were included in this study. Peritoneal CT attenuation values expressed in Hounsfield units (HUs) were measured on a workstation and investigated in relation to laboratory data obtained on admission, severity of illness, and hospital mortality.

**Results:**

Peritoneal CT attenuation values were significantly negatively correlated with the Acute Physiology and Chronic Health Evaluation II (p < 0.01, R^2^ = 0.17) and sequential organ failure assessment (SOFA) (p < 0.01, R^2^ = 0.30) scores. Peritoneal CT attenuation values of hospital nonsurvivors (n = 7, 12.4 ± 11.0 HU) were significantly lower than those of hospital survivors (n = 105, 34.3 ± 15.8 HU). There was a significant negative correlation between peritoneal CT attenuation values, serum C-reactive protein levels (p < 0.01, R^2^ = 0.11), and the time after the onset of abdominal pain (p < 0.01, R^2^ = 0.08). Multivariate analysis revealed that the SOFA score was significantly associated with peritoneal CT attenuation values.

**Conclusions:**

Evaluation of peritoneal CT attenuation values in patients with UGI perforation is simple and can be used to objectively assess the severity of peritonitis, which can serve as a reference for treatment strategies.

## Introduction

In recent years, conservative or endoscopic treatment has been increasingly selected as the treatment option for upper gastrointestinal (UGI) perforations owing to advances in diagnostic technology, treatment with antacids and antibiotics, and the accumulation of a large amount of evidence for non-operative management ^(1), (2), (3), (4)^. However, UGI perforation has a high mortality rate if treatment is delayed or if appropriate treatment is not performed ^(5), (6), (7)^. Therefore, it is necessary to quickly decide on the treatment strategy for UGI perforation, and it is important not only to measure individual clinical and laboratory data, but also to evaluate the severity of the disease from a systemic perspective using a simple and quick method. Various scoring systems have been established to assess the severity of the disease in patients in intensive care units ^(8), (9), (10), (11)^. However, existing grading systems for illness severity are not universally accepted and involve complex combinations of various test values that seem unsuitable for emergency situations.

Computed tomography (CT) is now an essential modality for imaging patients with acute abdomen ^(12)^. Multidetector-row CT (MDCT) images of UGI perforations are characterized by thickening of the intestinal wall, ascites, and abscess, as well as free air in the peritoneal cavity ^(13), (14)^. As peritonitis caused by UGI perforation is often accompanied by peritoneal hyperemia, edema, and inflammation, we believe that MDCT can be useful for evaluating the extent of these conditions. Previously, we demonstrated that peritoneal CT attenuation values are significantly lower in patients with colorectal perforation, septic shock, and nonsurvivors, reflecting the severity of peritonitis in patients with gastrointestinal perforation ^(15)^. Furthermore, we found that peritoneal CT attenuation values reflect the severity of peritonitis in patients who underwent emergency surgery for UGI perforation, although in a small number of cases ^(16)^. In the present study, we examined the value of peritoneal CT attenuation values as an index for the severity of peritonitis associated with UGI perforation.

## Materials and Methods

### Patients

A total of 119 consecutive patients were admitted to the Department of Surgery of the National Defense Medical College Hospital, from 2005 to 2023, for UGI perforations due to peptic ulcer with acute abdominal pain and free air detected on abdominal CT. This study excluded seven patients who only underwent CT scans with intravenous contrast agents. The systemic inflammatory response syndrome (SIRS) criteria ^(9)^, the sequential organ failure assessment (SOFA) score ^(10)^, and the Acute Physiology and Chronic Health Evaluation II (APACHE II) score ^(11)^ were used to assess the severity of the disease upon admission. The time after the onset of abdominal pain, laboratory data, and mortality were also determined from medical and nursing charts.

Of the 16 patients who received conservative treatment, eight underwent plain CT scans during conservative treatment, and the changes in peritoneal CT values were compared at admission and after conservative treatment (interval 3.9 ± 1.1 days).

### Evaluation of peritoneal CT levels

All studies were performed using a 64 or more MDCT without any contrast agent. Water and air calibrations were performed quarterly and weekly, respectively, to adjust the CT attenuation values. Peritoneal CT attenuation values were assessed by the author (HT) in a clinically blinded manner, as reported previously ^(15)^. Briefly, a region of interest of approximately 5 mm^2^ was selected, and three arbitrary points in the peritoneum adjacent to free air were measured. Data represent the average of radiodensity at three sites, and are expressed in Hounsfield units (HUs). Although the scanner model was updated during the study period, the peritoneal CT values remained consistent over time. CT attenuation values were evaluated by two experienced physicians. A statistically significant correlation was observed between the peritoneal CT assessed by the two investigators (HT and YY), blinded to clinical outcomes (p < 0.001).

### Statistical analysis

Data are expressed as mean ± standard deviation. Comparisons between the two groups were performed using either the Mann-Whitney U test or the chi-square test. The correlations between the two groups were analyzed using Spearman’s rank correlation test. Univariate and multivariate analyses were performed using a Cox proportional hazards model. All statistical analyses were performed using JMP Pro v.17.0.0 (SAS Institute, Inc., Cary, NC, USA). The ability of the scoring systems to predict hospital mortality was assessed using the area under the receiver operating characteristic (ROC) curve (AUC). Differences between areas were compared to determine the discriminative power of scores. Data were analyzed using a statistical software package (MedCalc Software, Mariakerke, Belgium). Statistical significance was set at p < 0.05.

## Results

Gastric and duodenal perforations were diagnosed in 25 and 87 patients, respectively (Supplementary Table 1). The average age of patients with gastric perforation was significantly higher than that of patients with duodenal perforation (69.5 ± 11.3 vs. 60.9 ± 18.5 years, p < 0.05). Patients with gastric perforation had more frequent hospital mortality (16% vs. 3%, p < 0.05) and higher APACHE II score (21.3 ± 4.3 vs. 19.0 ± 4.0, p < 0.05). There were no differences in peritoneal CT attenuation values when compared to the body mass index, hemoglobin level, and hematocrit level (Table 1). Peritoneal CT attenuation values in older (>70 years old) and female patients were significantly lower than those in younger or male patients. Among the 81 patients whose ascites samples were sent for culture, peritoneal CT attenuation values in patients with positive culture results (i.e., bacteria or fungi) were significantly lower than those of patients with negative ascites culture results. Peritoneal CT attenuation values were significantly lower in nonsurvivors than in survivors.

**Table 1.** Correlation between the Peritoneal CT Levels and the Clinical Data

Variables		Number	Peritoneal CT Levels (HU)	p-Value
Age	≤70	66	36.8 ± 15.4	<0.01
	>70	46	27.4 ± 16.4	
Gender	M	78	35.1 ± 16.2	<0.05
	F	34	28.0 ± 15.9	
BMI	<22	72	33.1 ± 16.0	0.69
	≤22	40	31.8 ± 16.4	
Hemoglobin	<13	54	30.3 ± 16.8	0.09
	≤13	58	35.7 ± 14.9	
Hematocrit	<38.6	56	31.1 ± 16.8	0.22
	≤38.6	56	35.1 ± 15.2	
Treatment				
	Operation	96	31.9 ± 16.3	0.09
	Conservative	16	39.6 ± 16.5	
Culture test for ascites				
	Positive	48	28.3 ± 17.3	<0.05
	Negative	33	35.7 ± 14.6	
	Not examined	31	37.2 ± 15.3	
Hospital mortality			
	Yes	7	12.4 ± 11.0	<0.01
	No	105	34.3 ± 15.8	

BMI: body mass index; CT: computed tomography; F: female; M: male.

**Figure 1.**
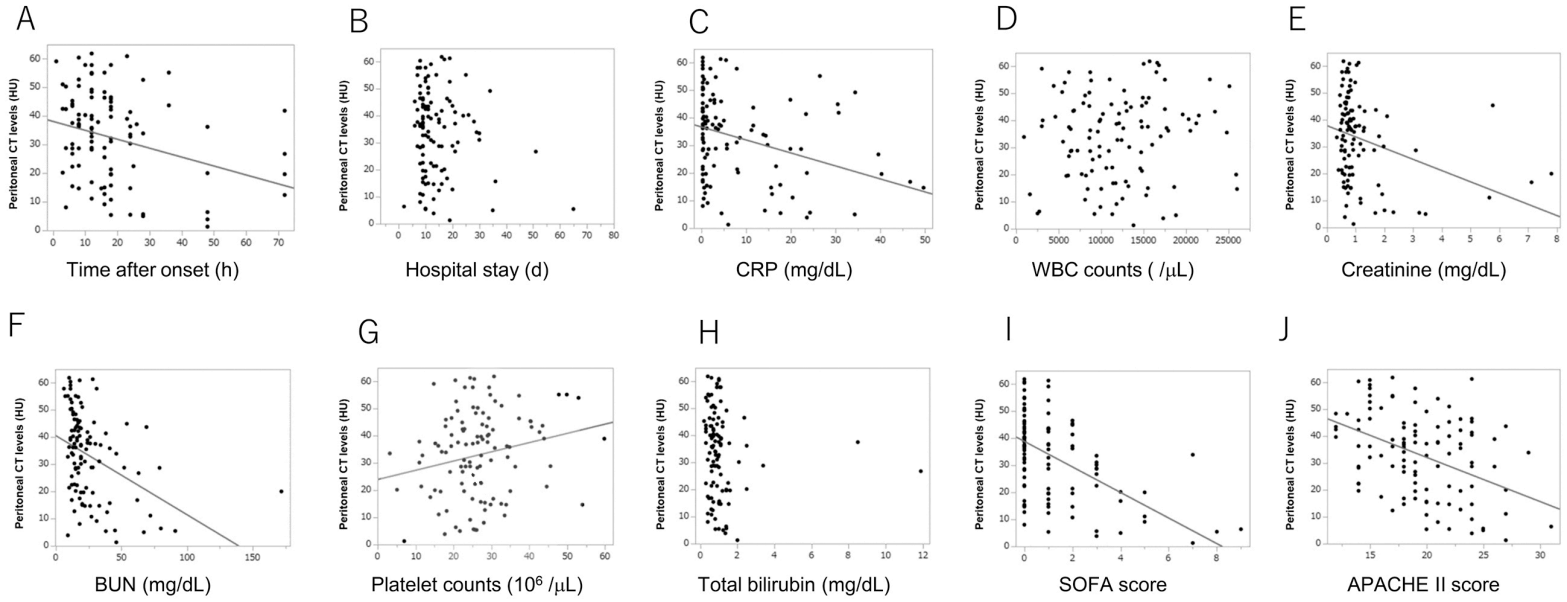
Correlations between peritoneal CT attenuation values and clinical parameters. The time after the onset of abdominal pain showed a significant negative correlation with peritoneal CT attenuation values (a), whereas the hospital stay showed no correlation with peritoneal CT attenuation values (b). There was a significant negative correlation between peritoneal CT attenuation values and serum CRP levels (c), whereas no correlation was observed between white blood cell counts (d). A statistically significant negative correlation was observed between the peritoneal CT attenuation value, serum creatinine level (e), and BUN (f), and a positive correlation was observed with platelet count (g); however, no correlation was observed with bilirubin levels (h). There were significant negative correlations between peritoneal CT levels and the SOFA (i) and APACHE II (j) scores. APACHE II: Acute Physiology and Chronic Health Evaluation II; CRP: C-reactive protein; CT: computed tomography; SOFA: sequential organ failure assessment.

The time after the onset of abdominal pain was significantly negatively correlated with peritoneal CT attenuation values (p < 0.01), whereas hospital stay (days) did not show any correlation with peritoneal CT attenuation values (p = 0.14) (Figure 1a and b). There was a significant negative correlation between peritoneal CT attenuation values and serum C-reactive protein (CRP) levels (p < 0.01), whereas no correlation was observed between peritoneal CT attenuation values and white blood cell counts (p = 0.21) (Figure 1c and d). A statistically significant negative correlation was observed between the peritoneal CT attenuation values, the serum creatinine levels (p < 0.01), and the blood urea nitrogen (BUN) levels (p < 0.01). Conversely, a positive correlation was observed with platelet counts (p < 0.05); however, no correlation was observed with bilirubin levels (p = 0.21) (Figure 1e-h).

We also investigated peritoneal CT attenuation values in terms of the severity of illness. Significant negative correlations were observed between peritoneal CT attenuation values and the SOFA and APACHE II scores (p < 0.01 for both) (Figure 1i and j). Subsequently, we examined the relationship between each factor of the SOFA score and peritoneal CT attenuation values (additional data are given in the Online Resource: Supplementary Figure 1). Of the six SOFA diagnostic scores, all except the coagulation score showed a significant negative correlation with peritoneal CT attenuation values.

In the univariate analysis, age, the SOFA score, and APACHE II score were significantly associated with peritoneal CT attenuation values. In the multivariate analysis, the SOFA score was significantly associated with peritoneal CT attenuation values (Table 2).

**Table 2.** Univariate and Multivariate Analyses of Factors that May Affect Peritoneal CT Levels.

Variables	Univariate analysis			Multivariate analysis		
	HR	95% CI	p-Value	HR	95% CI	p-Value
Age (>70)	1.70	1.15-2.51	<0.01	1.56	0.91-2.67	0.11
Sex (Male)	0.67	0.44-1.01	0.06	1.05	0.63-1.77	0.84
BMI (>22)	1.20	0.80-1.81	0.38	-	-	-
Site of perforation (Stomach)	0.69	0.44-1.11	0.11	-	-	-
Time after onset (<24 hours)	1.67	0.99-2.83	0.07	1.42	0.80-2.51	0.25
Culture test for ascites (Positive)	1.40	0.89-2.21	0.14	-	-	-
Hospital mortality (Yes)	7.01	3.11-15.8	<0.01	4.42	1.71-11.4	<0.01
SOFA score (>0)	2.02	1.37-2.96	<0.01	1.86	1.16-2.97	<0.01
APACHE II score (>19)	1.81	1.23-2.66	<0.01	1.35	0.78-2.34	0.28
SIRS (Yes)	1.28	0.86-1.92	0.22	-	-	-

APACHE II: Acute Physiology and Chronic Health Evaluation II; BMI: body mass index; CI: confidence interval; CT: computed tomography; HR: hazard ratio; SIRS: systemic inflammatory response syndrome; SOFA: sequential organ failure assessment.

**Figure 2.**
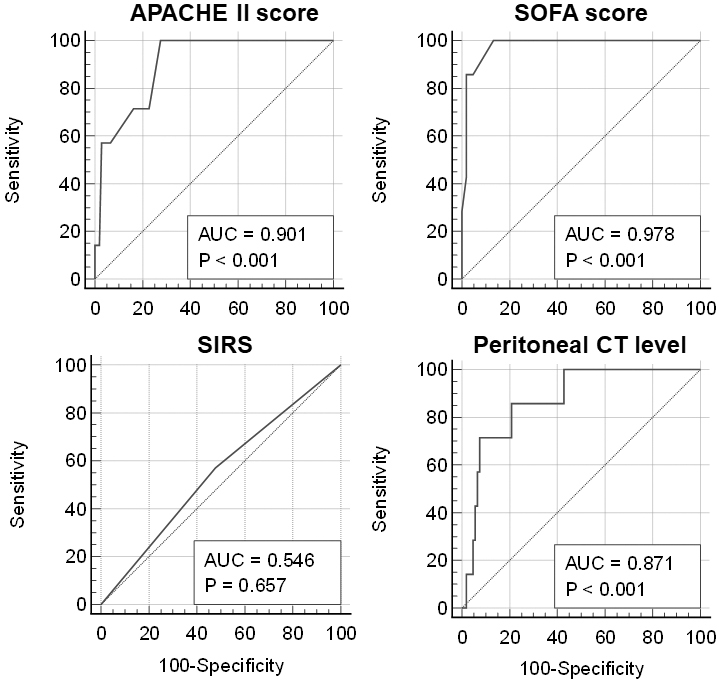
Receiver operating characteristic curves were used to discriminate between hospital survivors and nonsurvivors. APACHE II: Acute Physiology and Chronic Health Evaluation II; AUC: area under the receiver operating characteristics curve; CT: computed tomography; SIRS: systemic inflammatory response syndrome; SOFA: sequential organ failure assessment.

The ROC curves were used to evaluate the discriminative power of the scores for in-hospital mortality (Figure 2). The APACHE II score (AUC = 0.901), SOFA score (AUC = 0.978), and peritoneal CT attenuation values (AUC = 0.871) were significant in discriminating between in-hospital survivors and nonsurvivors. However, the AUC for the incidence of SIRS upon admission was not statistically significant.

As two patients had the second UGI perforation during the observation period, we assessed the correlation between severity and peritoneal CT attenuation values at the first and second perforation, respectively, and compared them in each case (Table 3). Even in the same case, the peritoneal CT attenuation values reflected the positivity of the ascites culture and the severity of the disease.

**Table 3.** Peritoneal CT Levels and Clinical Data in Patients with twice Upper Gastrointestinal Perforations.

		Patient 1	Patient 2
First time	CT value (HU)	5.4	0.4
	WBC (/μL)	11300	5500
	CRP (mg/dL)	4.5	39.0
	SOFA score	1	8
	APACHE II score	20	28
	Treatment	Local repair	Local repair
	Culture of ascites	*Candida glabrata*	*E. coli*, *E. faecalis*
	Hospital stay (d)	10	37
Second time	CT value (HU)	35.8	37.8
	WBC (/μL)	9600	10000
	CRP (mg/dL)	3.8	15.6
	SOFA score	1	0
	APACHE II score	18	18
	Treatment	Local repair	Conservative
	Culture of ascites	Not detected	-
	Hospital stay (d)	17	16

APACHE II: Acute Physiology and Chronic Health Evaluation II; CRP: C-reactive protein; CT: computed tomography; *E. coli*: *Escherichia coli*; *E. faecalis*: *Enterococcus faecalis*; SOFA: sequential organ failure assessment; WBC: white blood cell.

**Figure 3.**
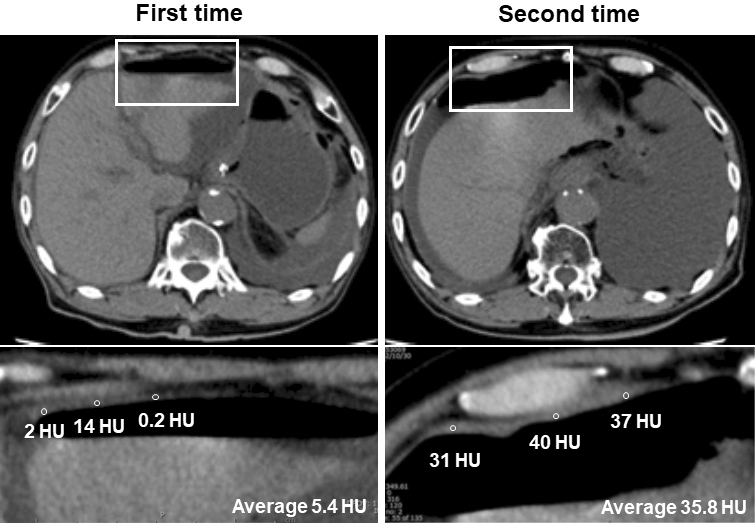
Representative abdominal plain CT images (upper panels) and peritoneal CT attenuation values (lower panels) in a patient with two duodenal perforations. The region of interest was created, and three arbitrary points on the peritoneum adjacent to free air were measured. Peritoneal CT levels (HU) were inserted. CT: computed tomography; HU: Hounsfield units.

The details of the clinical course in patient 1 were as follows: a man in his 70s had a history of esophageal cancer and underwent chemoradiotherapy. He had a left pleural effusion, but no cancer recurrence. The patient visited our hospital with a sudden onset of abdominal pain, and a CT scan performed 18 hours after the onset of symptoms revealed free air, with a peritoneal CT attenuation value of 5.4 HU (Figure 3). During emergency surgery, a perforation was found in the duodenal bulb, and a local repair was performed. *Candida glabrata* was detected in ascitic fluid cultures. Nine months after the first surgery, the patient returned to our hospital with sudden abdominal pain. A CT scan taken 12 hours after the onset of symptoms revealed free air, and the peritoneal CT attenuation value was 35.8 HU. A perforation was found in the duodenal bulb during emergency surgery, and local repair was performed. Ascites cultures revealed the absence of bacteria or fungi.

**Figure 4.**
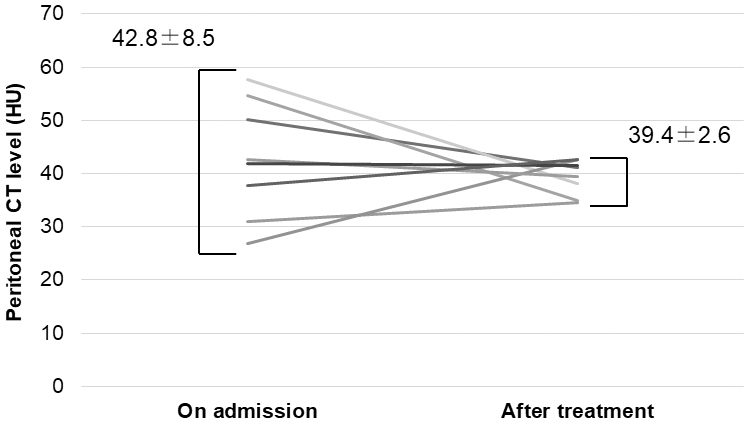
Peritoneal CT attenuation values in patients receiving conservative therapy for upper gastrointestinal perforations. CT: computed tomography; HU: Hounsfield units.

In the eight patients who received conservative treatment, the peritoneal CT attenuation values at the time of admission were relatively variable (42.8 ± 8.5 HU), and appeared to converge to around 39 HU after conservative treatment (39.4 ± 2.6 HU) (Figure 4).

## Discussion

In this study, we demonstrated that peritoneal CT attenuation values in patients with UGI perforations correlated with several parameters of systemic inflammation and organ dysfunction at admission, including CRP, serum creatinine, and BUN levels; microbial ascites; hospital mortality; and severity of illness scores; suggesting that peritoneal CT attenuation values could predict the severity of disease associated with UGI perforation.

CT is an effective modality for the diagnosis of gastrointestinal perforations and should be performed routinely in most patients with an acute abdomen. Moreover, recent advances in MDCT have allowed high-speed acquisition, thin-slice collimation, and reformatting of images in any plane with high spatial resolution ^(17)^. As critically ill patients frequently have kidney failure and cannot receive contrast agents, we evaluated peritoneal CT attenuation values using only plain CT images in this study. On plain CT, low peritoneal CT attenuation values can be associated with high fluid edema and inflammation, whereas high peritoneal CT attenuation values can reflect blood volume, such as congestion and hyperemia. In the present study, multivariate analysis revealed that peritoneal CT attenuation values were not affected by age, gender, or body mass index, but were affected only by in-hospital mortality and SOFA score. Using a workstation for electronic medical records, these findings can be easily and objectively evaluated without the need for special equipment.

To be clinically useful, a predictive model must demonstrate ease of use, precision, acceptance by data collection staff, and reproducibility ^(18)^. We showed that patients with severe peritonitis, as judged by higher APACHE II and SOFA scores, had lower peritoneal CT attenuation values in patients with UGI perforations. Furthermore, we have previously reported a significant positive correlation between peritoneal CT attenuation values evaluated by two physicians independently and in a blinded manner, indicating that this procedure is simple and provides an objective assessment with reproducible results ^(15)^. In addition, an association between peritoneal CT attenuation values and the severity of disease was observed in the same patients who presented with two UGI perforations. In this regard, we believe that the evaluation of peritoneal CT attenuation value may represent a promising parameter with several advantages over previously established scoring systems.

Peritoneal CT attenuation values varied widely among patients at the time of admission, but tended to concentrate around 40 HU in patients successfully treated with conservative treatment, suggesting that it may be possible to evaluate the appropriateness of conservative treatment in cases of UGI perforation by monitoring peritoneal CT attenuation values.

In conclusion, the evaluation of peritoneal CT attenuation values in patients with UGI perforations is simple and can objectively assess the severity of peritonitis. Furthermore, this procedure is useful for predicting clinical outcomes. While not intended to replace established severity scoring systems or clinical judgment, peritoneal CT attenuation values provide an imaging-based parameter that can aid rapid risk stratification, particularly in resource-limited or emergency settings. Given their strong correlation with SOFA and APACHE II scores and high AUC, we propose incorporating CT attenuation values as a supplementary tool to support clinical decision-making in ambiguous or rapidly evolving situations. A prospective study with a larger number of patients is required to establish the sensitivity and specificity of this method.

## Article Information

### Author Contributions

Material preparation, data collection, and analysis were performed by Risa Kariya, Seiichiro Fujishima, Takafumi Suzuki, Naoyuki Uehata, Yoshihisa Yaguchi, Keita Kouzu, and Hironori Tsujimoto. Hiroshi Shinmoto and Hideki Ueno supervised this project. The first draft of the manuscript was written by Hironori Tsujimoto and all authors commented on previous versions of the manuscript. All authors read and approved the final manuscript.

### Conflicts of Interest

None

### Acknowledgments

We would like to thank Editage (www.editage.jp) for English language editing.

### Data Availability

All data are available on request.

### Ethics Approval and Consent to Participate

All protocols were approved by the Institutional Review Board of the National Defense Medical College (Permission number: 4953). Written consent was obtained before the study.

### Patient Consent for Publication

All patients consented to this publication.

## Supplement

Supplementary Material Supplementary Figure 1
Correlation between the peritoneal CT attenuation values and each factor of the SOFA score.
Of the six SOFA diagnostic scores, cardiovascular (a), respiratory (b), renal (c), hepatic (d), and neurologic (f) scores had a significant negative correlation with peritoneal CT values. The coagulation score did not have a significant correlation with peritoneal CT values (e).
CT: computed tomography; SOFA: sequential organ failure assessment.
Supplementary Table 1. Clinical data in patients with upper gastrointestinal perforation.
APACHE II: Acute Physiology and Chronic Health Evaluation II; BMI: body mass index; CT: computed tomography; F: female; HU: Hounsfield units; M: male; SOFA: Sequential Organ Failure Assessment.
