# Supplementary material for: Predictive Impact of Peritoneal Computed Tomography Attenuation Values for the Severity of Upper Gastrointestinal Perforation
Source: JMA J. 2025 Sep 12;8(4):1293–300. doi: 10.31662/jmaj.2025-0189 (PMC12598265; doi:10.31662/jmaj.2025-0189)
Supplement: Supplementary Material — Supplementary Figure 1 Correlation between the peritoneal CT attenuation values and each factor of the SOFA score. Of the six SOFA diagnostic scores, cardiovascular (a), respiratory (b), renal (c), hepatic (d), and neurologic (f) scores had a significant negative correlation with peritoneal CT values. The coagulation score did not have a significant correlation with peritoneal CT values (e). CT: computed tomography; SOFA: sequential organ failure assessment. Supplementary Table 1. Clinical data in patients with upper gastrointestinal perforation. APACHE II: Acute Physiology and Chronic Health Evaluation II; BMI: body mass index; CT: computed tomography; F: female; HU: Hounsfield units; M: male; SOFA: Sequential Organ Failure Assessment. [file 2433-3298-8-4-1293-s001.pdf]

sTable 1 Clinical data in patients with upper gastrointestinal perforation

		All patients N=112	Stomach N=25	Duodenum N=87	p value
Age	(mean±SD)	62.8±17.5	69.5±11.3	60.9±18.5	<0.05
Gender	M: F	78: 34	14:11	64: 23	0.09
BMI		17.0±14.7	20.3±3.2	21.0±2.8	0.33
Time after onset (h)					
		17.0±14.7	16.8±11.3	18.6±16.0	0.88
Culture test for ascites					
	Positive	48	8	40	
	Negative	33	6	27	0.12
	Not examined	31	11	20	
Hospital stay (d)		16.1±21.7	15.5±8.1	16.3±24.3	0.13
Hospital mortality					
	Yes	7	4 (16%)	3 (3%)	0.02
	No	105	21 (84%)	84 (97%)	
SOFA score		1.30±2.0	1.72±2.25	1.10±1.77	0.32
APACHE II score		19.5±4.1	21.3±4.3	19.0±4.0	0.02
Peritoneal CT level (HU)		33.0±16.4	33.9±20.8	32.7±15.0	0.52

M: male, F: female, BMI: body mass index, SOFA: sequential organ failure assessment score, APACHE II: acute physiology and chronic health evaluation II score, CT: computed tomography, HU: Hounsfield units

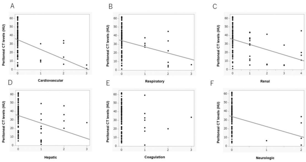
A
Peritoneal CT levels (HU)
Cardiovascular
B
Peritoneal CT levels (HU)
Respiratory
C
Peritoneal CT levels (HU)
Renal
D
Peritoneal CT levels (HU)
Hepatic
E
Peritoneal CT levels (HU)
Coagulation
F
Peritoneal CT levels (HU)
Neurologic
